# Applications of Limonene in Neoplasms and Non-Neoplastic Diseases

**DOI:** 10.3390/ijms26136359

**Published:** 2025-07-01

**Authors:** Katarzyna Rakoczy, Natalia Szymańska, Jakub Stecko, Michał Kisiel, Monika Maruszak, Michał Niedziela, Julita Kulbacka

**Affiliations:** 1 Faculty of Medicine, Wroclaw Medical University, 50-367 Wroclaw, Poland; katarzyna.rakoczy@student.umw.edu.pl (K.R.); natalia.szymanska@student.umw.edu.pl (N.S.); michal.kisiel@student.umw.edu.pl (M.K.); monika.maruszak@student.umw.edu.pl (M.M.); michal.niedziela@student.umw.edu.pl (M.N.); 2 Student Research Group No. K148, Faculty of Pharmacy, Wroclaw Medical University, Borowska 211A, 50-556 Wrocław, Poland; 3 Department of Molecular and Cellular Biology, Faculty of Pharmacy, Wroclaw Medical University, Borowska 211A, 50-367 Wrocław, Poland; 4 Department of Immunology and Bioelectrochemistry, State Research Institute Centre for Innovative Medicine, LT-08406 Vilnius, Lithuania

**Keywords:** limonene, breast cancer, hepatocellular carcinoma, diabetes mellitus, neurodegenerative diseases

## Abstract

Plants produce an extensive repertoire of secondary metabolites, developed over evolutionary time to support survival. Among these, D-limonene, a monoterpene exuded by citrus fruits, has demonstrated a broad range of pharmacological activities. This review elucidates limonene’s biological versatility, spanning antioxidant, anti-inflammatory, antitumor, antidiabetic, neuroprotective, and gastroprotective domains. Synthesizing data from both preclinical and early-phase clinical research, we explore its molecular mechanisms, ranging from reactive oxygen species mitigation and apoptosis induction to metabolic remodeling and neurotransmitter modulation. Special attention is given to limonene’s emerging role in oncological therapeutics, notably in breast and liver cancers, and its capacity to ameliorate pathophysiological hallmarks of diabetes and neurodegeneration. Its low toxicity and high bioavailability support its potential as a safe adjunct or alternative in phytotherapy. This review advocates for continued investigation into limonene’s translational potential across a spectrum of neoplastic and non-neoplastic diseases.

## 1. Introduction

**Figure 1:**
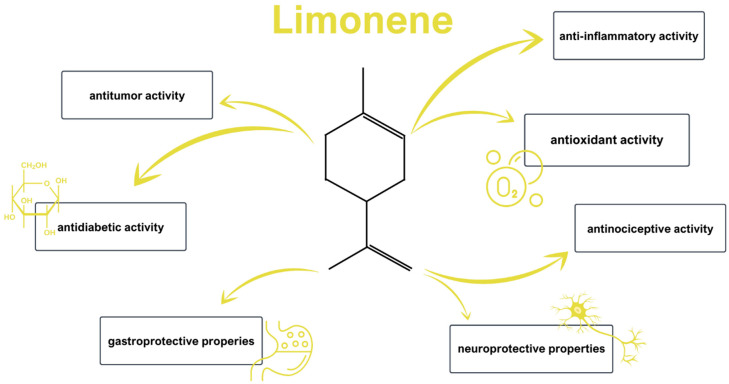
Biological activity of D-limonene: a variety of potential applications in medicine [3,4,5,6,7,8,9,10,11].

Biology abounds with examples of sophisticated defensive strategies which enable varied organisms to confront environmental stress in ways that are a derivative of their morphology. In the case of plants, sessility poses a significant challenge, as they cannot escape predators, competitors, or any adverse conditions, forcing them to rely on other means for survival. Millions of years of evolutionary pressure have resulted in the development of effective defense mechanisms, including the production of secondary metabolites [1]. Among the chemical abundance and complexity of those products, terpenes constitute a significant group. D-limonene or 4-isopropenyl-1-methylcyclohexene (C_10_H_16_) is the simplest monocyclic monoterpene that can be found in citrus plants, such as lemons, limes, oranges, tangerines, and grapefruits. Due to its remarkable fragrance properties, it is widely used as an ingredient in the production of essential oils, perfumes, soaps, foods, and beverages [2]. In the medical world, phytochemicals are famous for their noteworthy biological activities [3,4,5,6,7,8,9,10,11], which include antioxidant, anti-inflammatory, antinociceptive [12,13], antitumor [14], antidiabetic, gastroprotective, and neuroprotective properties (Figure 1). These effects have been demonstrated across diverse in vitro and in vivo studies, suggesting their potential for therapeutic application in both neoplastic and non-neoplastic disorders [15,16,17,18,19].

Importantly, implementing chemicals of natural origin, such as limonene, in combination with existing treatment lines has the potential to enhance therapeutic effects. For instance, combining limonene with tamoxifen increases the anticancer efficacy by inducing apoptosis in MCF 7 BC cells [20]. Among its other fascinating effects, the antidiabetic activity of limonene is based on its ability to inhibit the glycation process. The suggested mechanism assumes that the protein structure is stabilized through hydrophobic interactions, as in the presence of limonene, a decrease in protein unfolding is observed [8,21]. When it comes to the gastrointestinal tract, limonene can be used as a dissolving agent for gallstones [9] and can also relieve symptoms of gastroesophageal reflux di0sease (GERD) [10]. In vivo research confirms that limonene exhibits promising properties in peptic ulcer disease, as it causes an increase in gastric mucus, thus neutralizing H+ in the gastric juice. This occurs through increasing cell proliferation, angiogenesis, and production of PGE2 [22,23]. The neuroprotective potential of limonene has been demonstrated in different neurodegenerative diseases (NDs), including multiple sclerosis, stroke, epilepsy, Alzheimer’s disease (AD), and anxiety. The antioxidant and anti-inflammatory properties of the discussed monoterpene appear highly desirable, as neuroinflammation and oxidative stress are hallmarks of NDs [24]. Recent research suggests the nutritional use of products containing limonene and indicates the need for preclinical and clinical studies that examine limonene as an alternative or complementary phytomedicine [15]. Despite these promising findings, a comprehensive synthesis that consolidates limonene’s mechanisms of action across multiple disease areas is currently lacking. Moreover, its translational relevance, clinical data, and synergistic potential in combination therapies (e.g., with tamoxifen in breast cancer) remain underexplored. The motivation for this review arises from this gap and aims to systematically examine D-limonene’s biological functions across oncology, metabolism, neurology, and gastroenterology, and to critically assess its translational promise. We aim to highlight both preclinical findings and early clinical trials, providing a unified understanding of how this single compound may serve as a multi-target phytotherapeutic candidate. The novelty of this manuscript lies in its integrative scope, bridging traditionally siloed research domains to outline a consolidated view of limonene’s therapeutic versatility.

## 2. Applications of Limonene in Neoplasms

### 2.1. Breast Cancer

BC constitutes a major global health concern, representing the most prevalent malignancy among women and a leading contributor to cancer-related mortality [25,26]. Epidemiological data from 2022 indicate that BC was the second most frequently diagnosed cancer worldwide, with an estimated 2.3 million new cases [25]. Despite significant advancements in diagnostic modalities and therapeutic interventions, BC remained the fourth leading cause of cancer-related deaths, accounting for over 665,000 deaths globally [25]. These statistics underscore the persistent burden of the disease and highlight the urgent need for innovative preventive and therapeutic strategies.

D-limonene, a monocyclic monoterpene abundantly present in citrus essential oils, exhibits broad-spectrum anticancer activity through multiple, well-conserved molecular mechanisms [2]. Its antineoplastic effects are primarily mediated via the induction of apoptosis, disruption of mitochondrial integrity, and modulation of key oncogenic signaling cascades [27].

D-limonene influences the expression of pro- and anti-apoptotic proteins, promotes cytochrome c release, and activates caspase-dependent pathways, thereby triggering programmed cell death in various cancer cell types [27]. Additionally, it interferes with the Ras/Raf/MEK/ERK and PI3K/Akt signaling axes—pathways commonly associated with proliferation, survival, and chemoresistance—contributing to its cytostatic and pro-apoptotic effects [27]. The compound also demonstrates antiangiogenic and antioxidant properties, suggesting a pleiotropic mode of action across diverse tumor models [2].

In BC specifically, D-limonene targets key molecular pathways involved in tumor survival, cell cycle regulation, and apoptosis. In estrogen receptor-positive MCF-7 cells, D-limonene treatment significantly increases the expression of Bcl-2-associated X protein (Bax) and p53 while downregulating Bcl-2, inducible nitric oxide synthase (iNOS), and COX-2, collectively promoting mitochondrial dysfunction and caspase-mediated apoptosis [28]. These effects are further supported by cell cycle analyses showing enhanced late-phase apoptosis and suppressed G1/S transition [28]. Moreover, formulation strategies, such as encapsulation in chitosan nanoparticles, improve the compound’s bioactivity and delivery. In triple-negative BC (MDA-MB-468) cells, the IC_50_ of free limonene was reported to be 985.00 μg/mL, whereas its encapsulation in chitosan nanoparticles (LimChiNPs) significantly reduced the IC_50_ to 650.70 μg/mL. This indicates a moderate enhancement in cytotoxic efficacy upon nanoformulation. However, it remains notably less potent than Citrus sinensis oil-loaded nanoparticles (CSChiNPs), which achieved an IC_50_ of 23.65 μg/mL under the same experimental conditions [29].

Though preclinical studies have demonstrated D-limonene’s robust antiproliferative activity, evidence from clinical trials remains limited. A presurgical window trial by Miller et al. (2015) investigated the biological effects of D-limonene in women newly diagnosed with early-stage operable BC who were scheduled for tumor resection [30]. The study aimed to evaluate limonene’s systemic activity and its potential as a chemopreventive agent during the short interval between diagnosis and surgery [30]. Forty-three women received oral D-limonene at 2 g/day for 2 to 6 weeks prior to surgery. Paired plasma samples from 39 participants underwent metabolomic profiling, revealing significant changes in 72 of 397 identified metabolites. Notable alterations included decreased adrenal and gonadal steroid sulfates, and increased levels of bile acids, collagen breakdown products, and markers of altered glucose and lipid metabolism, indicative of mitochondrial stress and energy reprogramming [30]. Importantly, 47 metabolite changes significantly correlated with reduced cyclin D1 expression in tumor tissues, a critical regulator of cell cycle progression, which was lowered by an average of 22% [30]. These findings suggest that D-limonene exerts antiproliferative effects through metabolic remodeling and support its ability to reach and act within breast tissue [30]. While limited by sample size and semi-quantitative biomarker analysis, the study provides compelling evidence for limonene’s in vivo bioactivity and encourages further evaluation in placebo-controlled prevention trials. A phase I study by Vigushin et al. administered oral D-limonene at escalating doses up to 8 g/m^2^/day and reported a partial tumor response, defined as a ≥50% reduction in tumor size, in one BC patient, sustained for 11 months [14]. However, a subsequent phase II trial in patients with advanced BC did not observe measurable tumor responses, potentially due to late-stage disease and small sample size. These early-phase clinical data, along with findings from the metabolomic study by Miller et al., suggest that D-limonene is well-tolerated and biologically active in humans, particularly as a chemopreventive or neoadjuvant agent. Collectively, these findings underscore the need for larger placebo-controlled efficacy trials to confirm clinical benefits and elucidate mechanisms of action [14,20]. Recent studies have also investigated D-limonene’s potential in combination with existing therapies, particularly tamoxifen, to enhance therapeutic efficacy in estrogen receptor-positive (ER+) BC [20]. Mandal et al. (2023) demonstrated that co-administration of D-limonene with tamoxifen in MCF-7 cells led to a significantly greater reduction in cell viability and colony formation compared to monotherapy. Mechanistically, the combination induced apoptosis more robustly, evidenced by increased nuclear fragmentation and upregulation of pro-apoptotic Bax alongside suppression of anti-apoptotic Bcl-xL expression [20]. Furthermore, this dual therapy augmented intracellular reactive oxygen species production and promoted cell cycle arrest predominantly at the G1 phase via the modulation of cyclin D1 and B1 [20]. Notably, the migration capacity of MCF-7 cells was also markedly inhibited under the combined regimen, suggesting potential to curb metastatic progression [20]. These findings underscore the synergistic benefits of combining natural agents, like D-limonene, with endocrine therapy to overcome resistance and enhance clinical outcomes in ER+ BC treatment.

### 2.2. Hepatocellular Carcinoma

Liver cancer is a leading cause of mortality, ranking as the sixth most prevalent cancer by incidence and the third by number of deaths [25]. HCC represents the most common form of primary liver cancer; thus, it is often diagnosed at an advanced stage, limiting the effectiveness of curative treatments, like surgery or transplantation [31]. Given the poor prognosis and limited options for advanced cases, there is growing interest in identifying natural compounds with anticancer properties, such as limonene, that may offer new therapeutic strategies. Limonene has been shown to promote apoptosis in HCC cells by modulating the expression of key regulatory proteins [28]. Specifically, limonene upregulates pro-apoptotic factors, such as Bax and p53, while downregulating anti-apoptotic proteins, like Bcl-2, PTGS2 (COX-2), and iNOS [28]. The shift in the Bax/Bcl-2 ratio leads to mitochondrial dysfunction, cytochrome c release, and activation of caspases, activating programmed cell death [32]. A study by E. I. Salim et al. demonstrated that the combination of metformin and limonene resulted in enhanced apoptosis in HCC cells, underscoring the potential of combination therapies for improving treatment outcomes [28].

Oxidative stress plays a pivotal role in HCC development [33]. Diethylnitrosamine (DEN) and 2-acetylaminofluorene (2-AAF) can both cause oxidative stress, which can lead to liver and lung cancer [34]. Limonene therapy resulted in a decrease in lipid peroxidation levels and an increase in the level of glutathione, a major antioxidant that helps protect cells from damage [34]. Moreover, the activity of antioxidant enzymes (SOD and glutathione peroxidase (GPx)) was improved, indicating that the body’s natural defense system was functioning better again [34]. In conclusion, limonene treatment has been shown to mitigate liver damage caused by DEN/2-AAF exposure by reinforcing the antioxidant defenses in hepatic cells [34].

### 2.3. Lung Cancer

Lung cancer is both the most common and the deadliest cancer in the world. According to the International Agency for Research on Cancer (IARC), in 2022, almost 2.5 million people were diagnosed with lung cancer, and more than 1.8 million of them died [35]. Therefore, numerous studies have been conducted to develop new anticancer therapies and treatment strategies. The effect of limonene on lung cancer cells varies in the mechanism of action and its influence on different cell lines. Although the exact mechanism of its anticancer properties is still unknown, D-limonene is considered to induce apoptosis and inhibit disease progression through the regulation of lipid metabolism [6,36,37].

**Figure 2:**
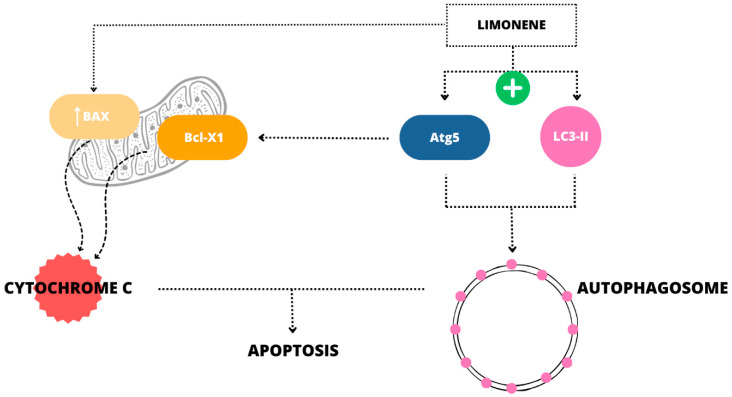
Apoptosis induced by limonene in lung cancer cells [6].

Autophagy is a process that degrades and recycles cellular components to maintain intracellular homeostasis [38]. Limonene induces the activation of autophagy, which subsequently leads to apoptosis (i.e., programmed cell death), thereby suppressing tumor growth [6]. Research indicates that limonene treatment leads to the accumulation of autophagosomes and increased expression of autophagy-related proteins, such as LC3-II and Atg5 [6]. Atg5 is not only involved in the formation of autophagosomes, but also binds to Bcl-X1 and promotes the secretion of cytochrome c, which activates apoptosis [39]. Limonene-induced apoptosis is also a result of upregulation of the expression of Bax, which leads to mitochondrial outer membrane permeabilization, releasing cytochrome c and activating caspases, culminating in programmed cell death (Figure 2) [6].

Cancer cells often reprogram lipid metabolic pathways to meet increased demands for energy, membrane biosynthesis, and signaling molecule production [40]. In lung cancer, dysregulated lipid metabolism contributes to tumor development [41,42]. In the study of Li et al., six genes were identified as being associated with lung adenocarcinoma (LUAD) patients’ prognosis, suggesting that targeting lipid metabolic pathways could be a promising therapeutic strategy [41].

**Figure 3:**
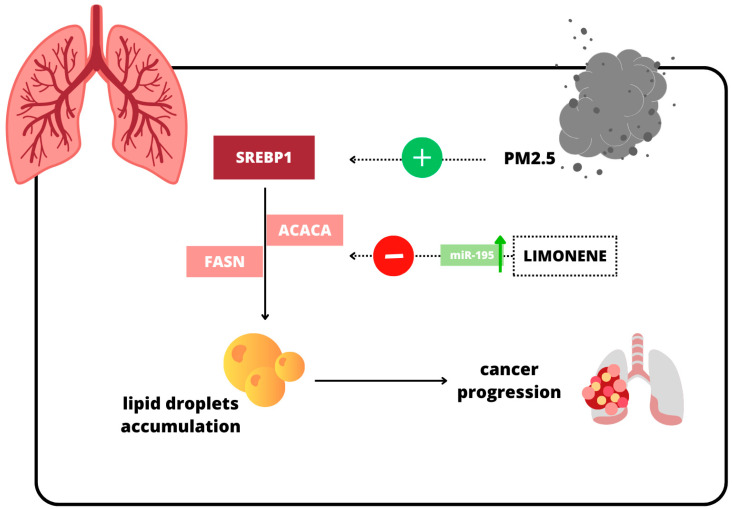
Limonene disrupts lipid metabolism and suppresses cancer progression [36]. Symbol key clarification: green “+”, activation; red “–”, inhibition; green “↑”, increased expression; dotted arrow, regulatory influence; solid arrow, metabolic/phenotypic flow. Limonene induces miR-195 (green “↑”), and miR-195 in turn inhibits SREBP1 (red “–”), thereby blunting the lipogenic cascade.

Exposure to particulate matter (PM2.5) has been linked to increased lipid droplet accumulation in lung tissues as PM2.5 exposure leads to the activation of Sterol Regulatory Element-Binding Protein 1 (SREBP1), a transcription factor that regulates lipid biosynthesis [36]. Upon activation, SREBP1 translocates to the nucleus. It upregulates the expression of lipogenic enzymes, such as fatty acid synthase (FASN) and acetyl-CoA carboxylase (ACACA), thus enhancing de novo lipogenesis, resulting in increased lipid droplet formation within lung cancer cells [36]. Limonene inhibits lipid droplet accumulation through the upregulation of microRNA-195 (miR-195), which suppresses the expression of SREBP1, FASN, and ACACA (Figure 3) [36].

Currently, there are no clinical trials specifically investigating limonene as a treatment for lung cancer. While preclinical data suggest potential benefits, these have not yet been translated into dedicated clinical studies aimed at treating the disease. However, there is one trial designed to investigate the chemopreventive effect of limonene in inhibiting the occurrence or progression of ground glass pulmonary nodules [43]. The primary objective is to determine whether limonene can serve as a safe and effective agent in preventing the development or progression of these nodules, thereby potentially expanding the therapeutic indications for limonene [43]. A major limitation in current lung cancer prevention strategies is the lack of effective methods for individuals who have never smoked and those at lower risk based on smoking history. Current screening guidelines are primarily tailored for individuals with a significant history of tobacco use [44]. The focus on smoking history as the primary risk factor leaves a substantial gap in addressing the needs of a growing population of lung cancer patients who do not fit this profile, such as those exposed to second-hand smoke or air pollution. A significant concern is the high rate of false-positive results, where the scan suggests the presence of lung cancer when none exists [44]. This can lead to unnecessary and often invasive follow-up tests, causing considerable anxiety for patients and increasing healthcare costs [44]. Limonene’s favorable safety profile and pharmacokinetic properties in humans, including cancer patients, provide a strong rationale for further clinical investigation into its potential role in lung cancer management [43,44]. However, additional research, including clinical trials, is necessary to evaluate its efficacy and safety as a therapeutic agent for lung cancer. While preclinical research shows D-limonene’s anticancer potential, the transition from preclinical findings to clinical application remains in its early stages. The existing clinical studies, though limited, provide encouraging signals of its safety and biological activity, particularly in a chemopreventive or neoadjuvant context for breast cancer, and point towards promising synergistic effects with conventional therapies. However, definitive evidence of its efficacy as a therapeutic agent for established tumors, either as monotherapy or in combination, remains to be established through larger, well-designed, placebo-controlled clinical trials. The focus should expand to strategically designed combination therapies and improved delivery methods to fully harness the therapeutic potential of this natural compound.

## 3. Applications of Limonene in Non-Neoplastic Diseases

### 3.1. Diabetes Mellitus and Other Metabolic Diseases

Diabetes mellitus is a widespread metabolic disease, posing a threat to an array of organs and systems due to numerous complications caused by persistent hyperglycemia. One of them is an elevated level of advanced glycation end products (AGEs) in the bloodstream, which, through their corresponding receptor, RAGE, and various subsequent signaling pathways, mediate molecular and metabolic changes. These pertain to the induction of insulin resistance, arteriosclerosis, nephropathy, neuropathy, retinopathy, cardiopathy, and the promotion of adipogenesis. Moreover, AGEs enhance the oxidation of LDL, which acts as a ligand for RAGE as well, not without metabolic consequences. Due to the myriad complications that AGEs cause, in recent years, they have been in the spotlight of diabetes research, especially regarding anti-AGE and RAGE-inhibiting agents. Aminoguanidine (AMG) was found to be one of them, yet its toxicity and high required doses resulted in withdrawal from phase III clinical trials [45]. In several studies, phytochemicals have emerged as a possible AGE counteragent, among which limonene gained recognition [8,46,47]. Across various rodent models of diabetes, D-limonene consistently lowered fasting glucose and HbA1c, improved lipid profiles, and enhanced antioxidant defenses (e.g., increased SOD, CAT, and GSH levels) [48,49,50,51,52,53,54,55,56,57,58]. Renal and hepatic parameters also improved, with a reduction in markers of oxidative stress and organ damage.

D-limonene has been shown to inhibit the formation of advanced glycation end products (AGEs) through multiple mechanisms, including the stabilization of protein structures and the prevention of α-helix to β-sheet transitions. Compared to aminoguanidine, a known antiglycation agent, limonene demonstrated comparable or superior efficacy at lower concentrations. These effects suggest its potential in mitigating glycation-related diabetic complications, such as nephropathy, cataract formation, and neuropathy [8,21,46,51]. A recurring finding across numerous studies is limonene’s ability to restore oxidative balance. It increases enzymatic antioxidants (SOD, CAT, and GPx) and non-enzymatic antioxidants (GSH, vitamins C and E), while reducing markers of lipid peroxidation (MDA, TBARS, and LOOH) [49,51,52,53,55,59,60]. Additionally, limonene reduces pro-inflammatory cytokines, such as TNF-α and IL-6, and increases IL-10, supporting its role as an anti-inflammatory phytochemical in diabetic models [56,57]. Limonene has demonstrated lipid-lowering effects, reducing serum LDL, triglycerides, and total cholesterol, while increasing HDL levels [49,50,51,52,53,55,56,58]. Mechanistically, these improvements are linked to activation of PPARα and PPARγ pathways, regulation of adipogenesis and lipogenesis, and decreased hepatic fat deposition. Notably, limonene also stimulates differentiation and glucose uptake in adipocytes, suggesting a role in counteracting insulin resistance and obesity-related metabolic dysfunction [50,61,62,63].

Hepatic markers are also significantly improved by limonene therapy in diabetic and high-fat diet models [49,52,54]. These hepatoprotective effects have led to the first exploratory, double-blind, placebo-controlled trial investigating limonene’s impact on type A metabolism-associated fatty liver disease (MAFLD) [64]. Notably, exhaled limonene levels have been proposed as a potential non-invasive biomarker to differentiate MAFLD cirrhosis from healthy liver function [65]. To facilitate comparison across a wide spectrum of preclinical studies, Table 1 summarizes key in vivo and in vitro findings related to D-limonene’s metabolic effects. This includes model types, dosages, and primary outcomes, such as glycemic control, lipid modulation, and antioxidant activity. While individual studies are referenced in the text, the table consolidates these data to support cross-study comparison, highlight recurring patterns, compare experimental conditions, and evaluate translational relevance, all of which are essential for guiding future preclinical design and clinical application of D-limonene in metabolic disorders.

Collectively, the studies summarized in Table 1 underscore several recurring findings: firstly, that D-limonene consistently lowers blood glucose and HbA1c levels in diabetic animal models, as well as enhances endogenous antioxidant enzymes, like SOD and CAT. Secondly, it improves lipid profiles by decreasing LDL and TG while raising HDL. Notably, the effective doses range from 50 to 300 mg/kg in rodent models, suggesting a relatively narrow therapeutic window. These consistent effects validate D-limonene’s potential as an anti-diabetic phytocompound, warranting translational exploration.

### 3.2. Gastrointestinal Diseases

Since 1976, the properties of limonene have been found to be beneficial in gastrointestinal diseases. Igimi et al. reported its use in vitro and in vivo as a gallstone dissolver twice. Administered through a choledochal catheter, 97% limonene solution dissolved retained cholesterol gallstones in the bile duct in at least half of 200 cases. Such an application seems promising as it would omit the need for a reoperation [9,67]. Limonene’s good litholytic activity was also described by Arrout et al. [68]. Moreover, this monoterpene proves itself useful in the reduction of intestinal inflammation, as reported by D’Alessio et al. and Kathem et al. [69,70]. D’Alessio described limonene’s protective effect on the epithelial barrier and its ability to lower serum concentrations of TNF-α and peripheral IL-6 in a rat model of colitis [69]. On the other hand, Kathem et al. showed a strong anti-inflammatory effect in mice with jejunal injury. Limonene attenuated the gene expression of TLR4, AP-1, and NF-κB in jejunal tissues and reduced iNOS expression as well as TNF-α, IL-1β, and COX-2 production. Furthermore, at a dose of 200 mg/kg, it amplified Nrf2 gene expression in jejunal tissues, exerting additional antioxidative action [70]. In line with this data is research by Yu et al. on the influence of limonene on ulcerative colitis in rats. Among the findings were the inhibition of TNF-α, IL-1β, IL-6, NF-κB, iNOS, COX-2, and TGF-βmRNA expression, and reduced MMP-2 and -9 mRNA expression. Moreover, SOD and GSH activities were increased and the ERK1/2 signaling pathway was activated, whereas PGE2 production was decreased. The importance of the latter is distinct, as mucosal PGE2 content, increased in ulcerative colitis patients, is interconnected with the degree of mucosal inflammation [71]. In a 2025 study by Senthil et al., limonene treatment increased the expression of occludin, claudin-1, ZO-1, and E-cadherin, molecules contributing to cellular cohesion. Additionally, a substantial reduction in β-glucose and 2-succinamate was detected, therefore suggesting limonene’s impact on intestinal epithelial cells’ glucose uptake and glutamate metabolism. Lastly, limonene’s CB1R antagonistic property, as stated in the paper, could effectively aid in the recovery of intestinal barrier damage [72].

Another use of limonene was reported by Moraes et al. and Souza et al., namely gastroprotection through local mucosal defense mechanisms and enhanced regeneration. Each research was conducted on gastric ulcers induced in rats and found reduced damage after limonene treatment [73,74]. In addition, in the study by Moraes et al., limonene improved the quality of the regenerated epithelial glandular structures, which is important in terms of avoiding relapses, and increased angiogenesis, a process essential for healing, in the lesion border. Interestingly, the increase in the epithelial regeneration height was comparable to that in cimetidine-treated rats [73]. Souza et al. described an increase in mucus production and higher preservation of gastric mucosa integrity. Furthermore, MPO activity, a biomarker of neutrophil infiltration, was reduced, GPx activity was increased, and an anti-inflammatory effect was observed through decreased levels of TNF-α, IL-6, and IL-1β and elevated IL-10 levels [74]. Gastric cancer prevention and mitigating properties of limonene were signaled by Lu et al. and Shen et al. [75,76]. Research by Lu et al. pointed to inhibition of tumor growth and metastasis as a result of limonene’s proapoptotic and antiangiogenic effects, the latter possibly through down-regulation of VEGF [75]. According to Shen et al., limonene can lessen 9 mRNA and MMP-2 expression levels by managing iNOS, PGE2, TGF-β, COX-2, and ERK1/2 signaling pathways, therefore diminishing disease progression and colonic mucosal damage [76].

### 3.3. Neurodegenerative Diseases

NDs, including AD, Parkinson’s disease (PD), Huntington’s disease, and amyotrophic lateral sclerosis (ALS), are a group of disorders characterized by the progressive loss of structure and function of neurons, ultimately leading to cognitive and motor impairments [77]. The main risk factor for NDs is older age; thus, as society is becoming older, finding an effective treatment against these conditions becomes a higher priority [77]. Limonene has shown promising potential in mitigating neurodegeneration through its neurotransmitter-modulating, anti-inflammatory, and antioxidant effects [4,78]. Limonene’s impact has been explored in several neurodegenerative conditions, with Alzheimer’s and Parkinson’s diseases appearing as the most studied.

**Figure 4:**
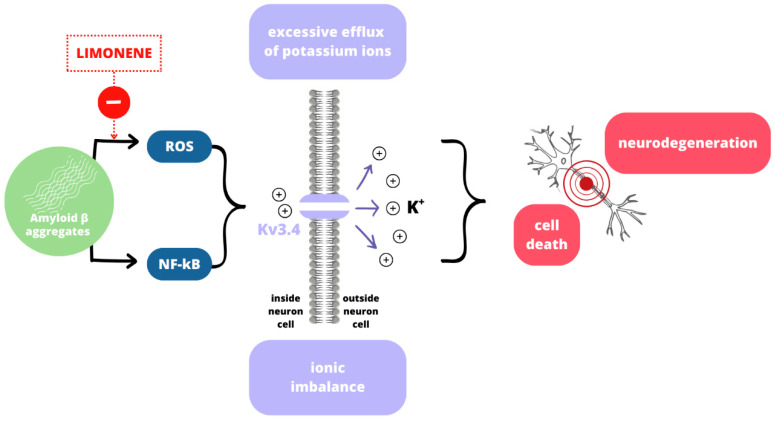
Aβ1-42 activation of Kv3.4 channels through ROS and NF-kB leads to ionic imbalance and neuron death [4]. Limonene decreases ROS production triggered by Aβ_1-42_ and limits the neurodegeneration process [4].

ROS play an important role in the development of NDs, as they are not only a cause but also a consequence of nervous system damage, leading to the formation of a detrimental positive feedback loop [79]. Oxidative stress can contribute to the development of amyloid beta (Aβ) and Tau proteinopathy [79]. The neuroprotective properties of limonene find expression in suppressing Aβ-induced cell death and decreasing ROS levels [4,16]. While the exact underlying mechanism remains unclear, several potential explanations have been proposed (Figure 4).

Kv3.4 plays a pivotal role in oxidative stress-related neural cell damage as an oxidation-sensitive channel involved in AD pathomechanism. Aβ1-42 oligomer exposure in primary cortical neurons selectively upregulates the activity of Kv3.4 potassium channels by the use of ROS mediation and transcription factor NF-kB activation [4,80]. Increased Kv3.4 activity leads to an excessive efflux of potassium ions from the neurons that provokes ionic imbalances, neuronal dysfunction, and cell death [4]. Limonene significantly decreases ROS production triggered by Aβ_1-42_ oligomers, thereby preventing Kv3.4 hyperactivity [4]. Oxidative stress is also increasingly recognized as a critical factor contributing to the degeneration of dopaminergic neurons in PD [81]. Limonene can enhance the activity and levels of endogenous antioxidant enzymes, such as SOD, CAT, and GSH, which are crucial for neutralizing ROS within the cell [82]. Similar to the interaction between Aβ and ROS in AD, α-synuclein creates a vicious cycle where inflammation promotes its aggregation, and α-synuclein, in turn, increases the expression of pro-inflammatory cytokines through microglia activation [83,84]. Limonene was initially identified as an anti-inflammatory factor in depression research, but these properties may also be useful in PD treatment [82,85]. Limonene’s anti-inflammatory properties are evident in suppression of pro-inflammatory cytokines, including tumor necrosis factor-alpha (TNF-α), interleukin-1 beta (IL-1β), interleukin-6 (IL-6), and interleukin-8 (IL-8) [82]. Limonene can also inhibit the activation of the NF-κB signaling pathway, a master regulator of inflammatory responses [82]. By targeting multiple aspects of the inflammatory cascade, limonene may help mitigate chronic neuroinflammation, thereby reducing neuronal damage and slowing disease progression in PD.

The anti-inflammatory properties of limonene could be beneficial in managing symptoms associated with multiple sclerosis (MS). Limonene increases the IL-10/IL-2 ratio, thereby enhancing levels of IL-10 [15]. However, limonene’s analgesic effects are even more prominent in MS. Limonene is suggested to activate the CB2 receptor and, along with other terpenes found in medical cannabis, may enhance the absorption of cannabinoids, which are used to alleviate various MS-related symptoms [15]. In fact, a clinical trial entitled ‘Behavioural Pharmacology of Orally Administered THC and D-limonene’ has been conducted to explore these effects. Limonene has been previously shown to influence the acute impact of vaporized THC, and this study seeks to determine if oral administration of limonene similarly modulates the acute effects of orally co-administered THC [NCT06378957].

Acetylcholinesterase (AChE) plays a crucial role in NDs by regulating levels of acetylcholine (ACh), a neurotransmitter essential for cognitive function, memory, and motor control [86]. The selective loss of cholinergic neurons in the basal forebrain, which project to memory-critical regions, such as the hippocampus and cortex, results in a substantial decline in acetylcholine levels [86].

Recent scientific investigations have explored the potential of limonene to modulate AChE activity. Given the significant role of cholinergic dysfunction and altered AChE activity in neurodegenerative disorders, the capacity of limonene to influence this enzyme warrants a closer examination of the underlying molecular biology and cellular mechanisms involved. In fact, an in vitro study demonstrated that the inhibitory activity of limonene is comparable to that of galantamine, a clinically used AChE inhibitor for AD [4]. Further insights into the molecular mechanism of limonene’s inhibition of AChE have been provided by molecular dynamics simulations, which have shown that limonene can bind to specific sites within the AChE enzyme, leading to alterations in the binding pocket of ACh [87]. This interaction is proposed to sterically hinder the binding of acetylcholine to the active site, thereby inhibiting the enzyme’s catalytic activity towards acetylcholine hydrolysis [87].

In PD, while the primary focus is on the loss of dopaminergic neurons in the substantia nigra, a growing body of evidence highlights the significant involvement of the cholinergic system in the disease’s pathophysiology, particularly in relation to cognitive impairment [88]. While direct evidence of limonene’s influence on AChE activity in PD models is less prominent in the reviewed literature, the established cholinergic involvement in PD warrants further investigation into this aspect.

## 4. Summary

D-limonene, the simplest of monocyclic monoterpenes, is a naturally occurring compound with diverse biological and therapeutic effects. This review traverses its diverse medicinal landscape, uncovering the way in which one molecule can simultaneously quell inflammation, scavenge free radicals, modulate metabolism, and steer apoptosis. Its therapeutic promise echoes across oncology, where it synergizes with tamoxifen in BC and fosters mitochondrial dysfunction in HCC. In metabolic disorders, limonene combats glycation, restores enzymatic equilibrium, and even stirs adipocyte differentiation. Limonene shows neuroprotective potential by mitigating oxidative stress in the central nervous system, as it tempers the excitotoxicity of amyloid and α-synuclein through antioxidant and anti-inflammatory pathways. The gastrointestinal tract also benefits from gallstone dissolution, as limonene aids in mucosal fortification. However, despite its low toxicity and promising preclinical data, the clinical translation of D-limonene remains limited. To fully realize its therapeutic potential, future research should adopt a more systematic approach. Specific areas warranting investigation include pharmacokinetics, bioavailability, and drug delivery strategies, such as nanoformulations, which have shown enhanced cytotoxicity in vitro. Additionally, a detailed safety profile, including long-term toxicity assessments, is critical for establishing its viability in clinical settings. Integrating these parameters into future trials will not only strengthen the translational bridge but also refine D-limonene’s potential as an integrative therapeutic agent across diverse disease spectra.

## Figures and Tables

**Table 1:** Overview of research papers on the antidiabetic and metabolic influences of limonene.

Author, Year	Model	Dose	Effects
Santiago et al., 2012 [49]	HFD-fed, L-NAME-treated rats	2% d- limonene diet	↓ fasting blood glucose, plasma insulin, HOMA-IR, pancreatic B-cell mass and hyperplasia, and B-cell nucleus ↓ SBP and HR ↓ lipid peroxidation byproducts ↑ GST and DT-diaphorase ↑ GSH, vitamin C, and vitamin E ↓ AST, ALT, and ALP ↑ TC, TG, and FFA ↓ hepatic enzymes: cytochrome P450, cytochrome b5, cytochrome P4502E1, NADPH-cytochrome P450 reductase, and NADH-cytochrome b5 reductase ↓ hepatic fat deposition and hepatosteatosis
Murali et al., 2012 [48]	STZ-induced diabetic rats	50 mg/kg, 100 mg/kg, 200 mg/kg	↓ blood glucose (maximum effect at 100 mg/kg) and HbA1c ↑ body weight, hemoglobin, and plasma insulin ↑ enzyme activity in hepatic tissue ↑ glycolysis, glycogenesis, pentose oxidative pathway, and glycogen content in liver ↓ gluconeogenesis
Jing et al., 2013 [50]	HFD-induced obese mice	0.6 g/kg	↓ blood glucose ↑ improved impaired glucose tolerance at 60 and 90 min ↓ plasma LDL-c and serum TG ↑ plasma HDL-c TC, body weight unaffected ↑ PPARα transactivity ↓ LXRβ signaling ↑ PGC-1α gene expression in WAT ↓ hepatic lipid deposition ↓ size of white and brown adipocytes
Murali et al., 2013 [59]	STZ-induced diabetic rats	100 mg/kg	↓ plasma glucose ↑ plasma insulin ↑ SOD, CAT, GPx, and GST activities ↑ GSH, vitamin C, vitamin E ↓ TBARS, LOOH, and CD - normal liver and kidney architecture
Panaskar et al., 2013 [8]	STZ-induced diabetic rats	A. marmelos extract: 150 µg/kg, 300 µg/kg Limonene: 10 µM, 50 µM, 100 µM	- potent antiglycative properties similar to AMG at an almost 20-fold lower concentration ↓ blood glucose ↓ progression of nephropathy and cataract formation in vivo
Joglekar et al., 2013 [21]	Bovine serum albumin	25 µM, 50 µM, 100 µM	- excellent protein glycation inhibitor - blocking transition of α-helix to β-sheet - stabilizing structure through hydrophobic interactions
Nalawade et al., 2014 [51]	STZ-induced diabetic rats Bovine serum albumin	20 mg/kg 25 µM, 50 µM, 100 µM	↓ fructosamine formation comparable to a tenfold greater AMG concentration ↑ SOD and CAT activity ↓ TBARS formation ↓ urine glucose, albumin, and creatinine - no blood glucose decrease in OGTT
Sharma et al., 2016 [60]	STZ-induced diabetic rats	100 mg/kg, 200 mg/kg	- attenuated behavioral and biochemical alterations of neuropathy ↑ CAT, GSH, and total protein levels ↓ nitrite and TBARS level - no hypoglycemic effect on healthy rats
Joglekar et al., 2017 [46]	Bovine serum albumin	25 µM, 50 µM, 100 µM	- reinforced mechanism of glycation inhibition - combination can reduce dosage of AMG by twenty times - combinatorial treatment of AMG and limonene inhibited AGE-related fluorescence and pentosidine formation
Bacanlı et al., 2017 [52]	STZ-induced diabetic rats	50 mg/kg	↓ plasma insulin levels ↓ GR, 8-OHdG, and MDA levels ↑ GSH, CAT, SOD, and GPx ↓ serum LDL, TC, and TG ↑ serum HDL ↓ AST and GGT ↓ DNA damage in blood, liver, and kidney cells
Soundharrajan et al., 2018 [62]	3T3-L1 preadipocytes	5 µM	- probable induction of differentiation and glucose uptake in 3T3-L1 preadipocytes - regulated adipogenesis and lipogenesis via induction of PPAR*γ*, C/EBP- α, C/EBP-β
Yilmaz et al., 2018 [66]	Alloxan-induced diabetic mice	0.15 mL/kg, 0.3 mL/kg, 0.6 mL/kg	- inflammatory effect (peak at 0.30 mL/kg) - no hypoglycemic effect
Kumar et al., 2020 [47]	STZ-induced diabetic rats, rat lenses	1–100 µM/mL, 13.49 µM/mL for lens incubation	↓ AR and AGE ↑ increased crystalline chaperone activity - delayed development of diabetic cataracts
Bagheri et al., 2021 [53]	Alloxan-induced diabetic rats	100 mg/kg	↓ serum glucose, creatinine, and urea ↑ GSH, mRNA of GPx, CAT, and SOD ↓ MDA, MPO, and NO
Valerii et al., 2021 [58]	HFD-fed mice	30 mg/kg 60 mg/kg	↓ fasting glycemia and TG ↓ weight gain ↓ HFD-associated liver steatosis ↑ liver PUFA levels
Shakeel et al., 2022 [54]	STZ-induced diabetic rats	300 mg/kg	↓ blood glucose, HbA1c ↑ serum insulin ↓ ALP, ALT, AST, and GGT ↑ albumin and total protein ↓ progression of liver degeneration
Han et al., 2023 [56]	HFD-fed, low-dose STZ diabetic atherosclerosis model in rats	200 mg/kg	↓ blood glucose ↓ cholesterol, TG, and LDL ↑ HDL/LDL ratio ↓ atherogenic index, morphological irregularities of the intima ↑ mnSOD and GSH ↓ 8-isoprostane ↓ TNF-α and IL-6 ↑ IL-10 ↑ expression of p-AMPK/ AMPK, SIRT1, and p-p65/p65 proteins
Lawal et al., 2023 [55]	Alloxan-induced diabetic rats	10 mg/kg, 5 mg/kg with 25 mg/kg vitamin E	↓ blood glucose level comparable to metformin ↑ body weight ↓ feed intake ↑ RBC and WBC levels ↑ hepatic glycogen levels ↓ MDA ↓ TC, TG, and LDH - ameliorative effect on β-cell of pancreas
Benchoula et al., 2024 [57]	HFD-induced type 2 diabetes-related obese zebrafish	5 mg/L, 20 mL/L	↓ fasting blood glucose and BMI - reverses changes in metabolites due to diabesity - reverses elevated expression of AKT

↓—decrease, ↑—increase.

## Data Availability

No new data were created or analyzed in this study.

## Abbreviations

**Abbreviation**	**Definition**
2-AAF	2-acetylaminofluorene
ACACA	Acetyl-CoA Carboxylase
ACh	Acetylcholine
AChE	Acetylcholinesterase
AD	Alzheimer’s disease
AGE	Advanced glycation end product
ALP	Alkaline phosphatase
ALS	Amyotrophic lateral sclerosis
ALT	Alanine aminotransferase
AMG	Aminoguanidine
AR	Aldose reductase
AST	Aspartate aminotransferase
ATG5	Autophagy-related gene 5
Aβ	Amyloid beta
Bax	Bcl-2-associated X protein
BC	Breast cancer
CAT	Catalase
CB2	Cannabinoid receptor type 2
CD	Conjugated dienes
COX-2	Cyclooxygenase-2
DEN	Ciethylnitrosamine
DT-diaphorase	NAD(P)H:quinone oxidoreductase
EGFR	Epidermal growth factor receptor
FFA	Free fatty acids
GPx	Glutathione peroxidase
GR	Glutathione reductase
GSH	Glutathione
GST	Glutathione S-transferase
HbA1c	Glycated hemoglobin
HDL	High-density lipoprotein
HFD	High-fat diet
HR	Heart rate
HOMA-IR	Homeostasis model assessment for insulin resistance
IC_50_	Half maximal inhibitory concentration
IL-1β	Interleukin-1 beta
IL-6	Interleukin-6
IL-8	Interleukin-8
IL-10	Interleukin-10
iNOS	Inducible nitric oxide synthase
lb-LDL	Large buoyant low-density lipoprotein
LC3-II	Microtubule-associated proteins 1A/1B light chain 3B
LDL	Low-density lipoprotein
LDL-c	LDL cholesterol
L-NAME	N(ω)-nitro-L-arginine methyl ester
LOOH	Lipid hydroperoxides
LUAD	Lung adenocarcinoma
LXRβ	Liver X receptor beta
MDA	Malondialdehyde
MCF-7	Human breast cancer cell line
mnSOD	Manganese superoxide dismutase, p-AMPK
MPO	Myeloperoxidase
MS	Multiple sclerosis
ND	Neurodegenerative disease
NF-κB	Nuclear factor kappa-light-chain-enhancer of activated B cells
NO	Nitric oxide
OGTT	Oral glucose tolerance test
8-OHdG	8-hydroxy-2′-deoxyguanosine
p-AMPK	Phosphorylated adenosine monophosphate-activated protein kinase
PD	Parkinson’s disease
PGC-1α	Peroxisome proliferator-activated receptor gamma coactivator 1-alpha
PI3K	Phosphoinositide 3-kinase
PPAR	Peroxisome proliferator-activated receptor
PUFA	Polyunsaturated fatty acid
RBC	Red blood cells
ROS	Reactive oxygen species
SOD	Superoxide dismutase
sd-LDL	Small dense low-density lipoprotein
SIRT1	Sirtuin 1
STZ	Streptozotocin
TBARS	Thiobarbituric acid-reactive substances
TC	Total cholesterol
TG	Triglycerides
TNF-α	Tumor necrosis factor-alpha
TRPA1	Transient receptor potential cation channel subfamily A member 1
WAT	White adipocyte tissue
WBC	White blood cell
